# H. William Schnaper - Life course journey of a true Mensch

**DOI:** 10.1007/s00467-021-04959-3

**Published:** 2021-03-17

**Authors:** Laurence Greenbaum, Victoria Norwood, Eileen Brewer, William Smoyer, Marva Moxey-Mims, Joseph Flynn, Barbara Fivush, Patrick Brophy, Brad Warady, Sandra Watkins, Isidro Salusky, Rick Kaskel

**Affiliations:** 1 Emory University School of Medicine, Atlanta, GA USA; 2 University of Virginia School of Medicine, Charlottesville, VA USA; 3 Baylor College of Medicine, Houston, TX USA; 4 Ohio State University School of Biomedical Science: The Ohio State University College of Medicine, Columbus, OH USA; 5 George Washington University Medical Center, Washington, DC USA; 6 University of Washington Seattle Campus: University of Washington, Seattle, WA USA; 7 Johns Hopkins University School of Medicine, Baltimore, MD USA; 8 University of Rochester Medical Center, Rochester, NY USA; 9 University of Kansas City: University of Missouri Kansas City, Kansas City, MO USA; 10 University of California Los Angeles, Los Angeles, CA USA; 11 Montefiore Medical Center, New York City, NY USA

H. William (Bill) Schnaper was the Irene Heinz Green and John LaPorte Given Chair in Pediatric Research and Tenured Professor and Vice Chair, Department of Pediatrics, Feinberg School of Medicine, Northwestern University. He was a graduate of the Baltimore Polytechnic Institute (1967) and received a B.A. from Yale University (1971) and his M.D. from the University of Maryland (1975). He trained in Pediatrics at New York’s Mount Sinai Hospital and was Chief Resident (1975–1978). It was there that he met a nurse named Maria and started a lifelong journey of love and family. He next served in the National Health Service Corps (1978–1980) and was a Senior Clinical Instructor in Pediatrics at Hahnemann Medical College (1979–1980). He then entered a fellowship in Pediatric Nephrology at St. Louis Children’s Hospital and the Washington University School of Medicine (1980–1982) under the mentorship of Alan Robson and supported by the National Kidney Foundation. He was a Research Fellow in Pathology at the Jewish Hospital of St. Louis under Carl W. Pierce during his training and through 1988. He received an NIH Clinical Investigator Award to examine the soluble immune response suppressor in nephrosis followed by an NIH R01 to examine inhibition of tumor cell growth by the lymphokine SIRS. He rose through the ranks as an Instructor, Assistant, and Associate Professor of Pediatrics at Washington University School of Medicine before moving to Washington, DC and joining the Faculty at Children’s National and George Washington University in 1990 as an Associate Professor. He also served as a Special Volunteer/Expert in the Laboratory of Developmental Biology at the NIH from 1990 to 1994.

In 1994, Bill joined the Faculty as an Associate Professor of Pediatrics at the Feinberg School of Medicine, Northwestern University Medical School, where he advanced to a tenured Professor in 2000. Bill assumed numerous leadership responsibilities including Director of the Fellowship Training Program, of the Research Career, Development and Physician Scientist Program, of the K and TL1 Postdoctoral Awards Programs of Northwestern University’s CTSA, and of the Pediatric Academic Affairs and Child Health Research Center (CHRC) and its Integrated Graduate and Fellowship Programs. As a faculty member, Bill was highly academically productive, with more than 170 peer-reviewed publications. He made seminal research contributions related to the immunologic basis and molecular pathogenesis of nephrotic syndrome, the role of cell signaling and tubulointerstitial injury in the development of renal fibrosis among other areas. In addition, he served as a mentor for over 60 students and trainees, ranging from high school, undergraduate college, medical school, graduate school, fellowship, post-doctoral training, and junior faculty. Bill served on and chaired more than 20 NIH and other scientific organization grant review committees and certainly facilitated the investigative careers of an entire generation of pediatric nephrologists.

As if these were not enough, Bill served on the Board of Directors of Children’s Memorial Medical Center. He also was Chair of the American Board of Pediatrics, Sub-Board of Pediatric Nephrology, later serving as a medical editor for the Sub-Board for many years, work that he continued to do through the late stages of his illness. Along with Bruder Stapleton, Dick Behrman, and Ted Sectish, he co-founded the Council of Pediatric Subspecialties. He played important roles in many scholarly publications, including being the Medical Editor of the journal *Nephrology* and on editorial boards for the *Journal of the American Society of Nephrology* and the *American Journal of Physiology* (Renal Physiology), as well as being a founding member and the longest member of the Editorial Board of the journal *Pediatric Nephrology*.

Distinguished service was part of Bill’s DNA, as exemplified by his service to the American Society of Pediatric Nephrology (ASPN) and the International Pediatric Nephrology Association (IPNA). He had the longest tenure ever on ASPN Council from 2000 to 2014 where he was Council Member (2000–2004), Secretary-Treasurer (2004–2008), President-Elect (2008–2010), President (2010–2012), and Past-President (2012–2014). He was the North American Regional Secretary of IPNA from 2004 to 2008 and Chair of the Scientific Organizing Committee for the 15^th^ IPNA Congress in New York City in 2010. He was also the organizer of the American Society of Nephrology’s “Why kidneys fail: translating basic mechanisms of disease progression in novel therapies” during Renal Week 2005. He was the recipient of the Jose Strauss M.D. Award for Career Academic Excellence in Pediatric Nephrology and received the ASPN’s highest honor, the Founder’s Award, in 2018.

During Bill’s long tenure on ASPN Council, he was challenged with multiple tasks including working with then President Lisa Satlin to transition the ASPN office to a permanent one with dedicated professional staffing. Along with Sandra Watkins and Sharon Andreoli, he conceptualized and initiated the Corporate Liaison Board (CLB) to strengthen ASPN’s fundraising efforts. Additionally, he heralded both the first ASPN Strategic Planning Initiative and Leadership Development Program along with Joseph Flynn. With Bill Smoyer, Marva Moxey-Mims, and others, he also started the ASPN Therapeutics Development Committee. He led an effort to have a Program Project Grant funded for ancillary studies to the NIH FSGS-Clinical Trial with four investigators receiving NIH funding. Finally and most importantly in terms of Bill’s legacy to ASPN, he participated in the founding of the ASPN John E. Lewy Advocacy Scholars Program, for which he became a mentor and nurtured advocacy skills of over two dozen junior pediatric nephrologists, helping to ensure that ASPN will be well-represented on Capitol Hill for the next generation.

Bill’s dedication to pediatric nephrology and academic medicine was only exceeded by his devotion to his family, including his wife Maria, daughter Adrienne, sons Michael and Owen, and his seven grandchildren. He coached soccer teams and mentored school science projects. Music was also an important part of Bill’s life. He played a variety of instruments—violin, bass guitar, and guitar—and performed in diverse settings such as a band while in college at Yale, his Synagogue choir, a local Klezmer band and singing the lyrics he had written for songs commemorating ASPN’s 50^th^ Anniversary. He was also a dedicated runner and completed the Chicago marathon.

So much has been said by so many colleagues about Bill and his impact on our community, and many of these are included here as an online supplement (see “Remembrances”). His remembrance will live on with the H. William Schnaper Honorary Lecture and his wonderful interview on the ASPN’s History Project site. At the end of the Memorial Service, everyone sang “Puff the Magic Dragon,” and I can only imagine Bill’s smiling face to us all as we said one “last goodbye.” (Rick Kaskel)



